# Healthcare workers’ knowledge, beliefs, and thoughts on improving hand hygiene practices in nursing homes

**DOI:** 10.3389/fpubh.2025.1633964

**Published:** 2025-11-19

**Authors:** Ida Hellum Sandbekken, Borghild Løyland

**Affiliations:** Department of Nursing and Health Promotion, Oslo Metropolitan University, Oslo, Norway

**Keywords:** hand hygiene, nursing home, healthcare workers, interview, nursing students

## Abstract

**Background:**

Hand hygiene adherence in nursing homes is too low to prevent transmission of healthcare-associated infections, and it is difficult to achieve long-lasting increased hand hygiene adherence. There is a need for more information about healthcare workers’ knowledge, beliefs, and routines in hand hygiene practices to better understand how to improve them.

**Methods:**

Five second-year students in a bachelor’s degree in nursing conducted nine interviews of healthcare workers as part of an intervention to increase hand hygiene adherence in a nursing home ward.

**Results:**

Four major topics emerged from the analyses of the transcript data: hand hygiene is important, inadequate knowledge, barriers to infection practices, and suggestions for improvement.

**Discussion:**

The healthcare workers knew that proper hand hygiene was important for preventing the spread of infections, but they had limited knowledge of when and how to perform it. They saw that other healthcare workers did not perform hand hygiene when it was required, but few of them wanted to correct or remind ‘the others’ of what was correct.

**Conclusion:**

The healthcare workers experienced stress, and tiredness and said it was challenging to balance providing good care for residents with proper hand hygiene. Nursing home leaders should ensure staff receive proper training, regular reminders, and access to hand hygiene resources.

## Introduction

Healthcare-associated infections are a major cause of suffering, hospital admissions, and death among residents in nursing homes (1). Proper hand hygiene is the most effective single preventive measure against the spread of infections (2). Although the importance of hand hygiene is widely recognized, compliance with guidelines remains too low to prevent infection transmission (3–5). There is a need to improve infection control practice and hand hygiene in nursing homes. Studies have shown that the effects of interventions for improving hand hygiene can diminish over time (6, 7).

Nursing homes in Norway are high care-requirement homes that provide nursing care and some treatment to their residents. Residents are mostly older adults with high rates of frailty and comorbidity (8). More than 80 percent of them have dementia (9), and their life expectancy is short, with a median survival of 2.2 years (8). In nursing homes, residents generally have their own rooms, but they share dining areas, living rooms, and sometimes bathrooms (10). The shared common spaces leads to close contact between residents (4, 11, 12). These factors place nursing home residents at particularly high risk of serious illness and death from infections (13, 14).

The World Health Organization (WHO) published the first global guidelines for hand hygiene practice for healthcare workers in 2009 (2). These guidelines include ‘My 5 moments for hand hygiene,’ five indications of when healthcare workers should conduct hand hygiene. The five moments are as follows: (1) before touching a patient, (2) before an aseptic/clean procedure, (3) after body fluid exposure risk, (4) after touching a patient, and (5) after touching patient surroundings (2).

Few studies have interviewed healthcare workers in long-term care facilities to obtain a deeper understanding of hand hygiene practices in this context. One study from the Netherlands interviewed 31 nurses and nurses’ assistants in care facilities for older adults, identified challenges in working hygienically while responding adequately to residents’ acute care needs (15). Similarly, a study from paediatric care facilities in New York found confusion among healthcare workers regarding hand hygiene recommendations, including the use of soap or sanitiser, and isolation precaution policies (16).

Nursing students learn greatly by being allowed to participate in research (5). Yet they are seldom included despite their a unique position through close connection with healthcare facilities during their practical sessions. In this study, students were actively involved by conducting interviews after thorough education.

To better understand how to change the behavior of healthcare workers, we need to know more about their knowledge, beliefs, and routines for hand hygiene practice. The aim of this study is to explore and identify healthcare workers in nursing homes’ knowledge and beliefs about infection prevention, their attitudes regarding their own and others’ practices, and their ideas and advice for possible improvements in infection prevention efforts. In addition, this study will evaluate the use of nursing students as interviewers of healthcare workers.

## Methods

**Figure 1:**
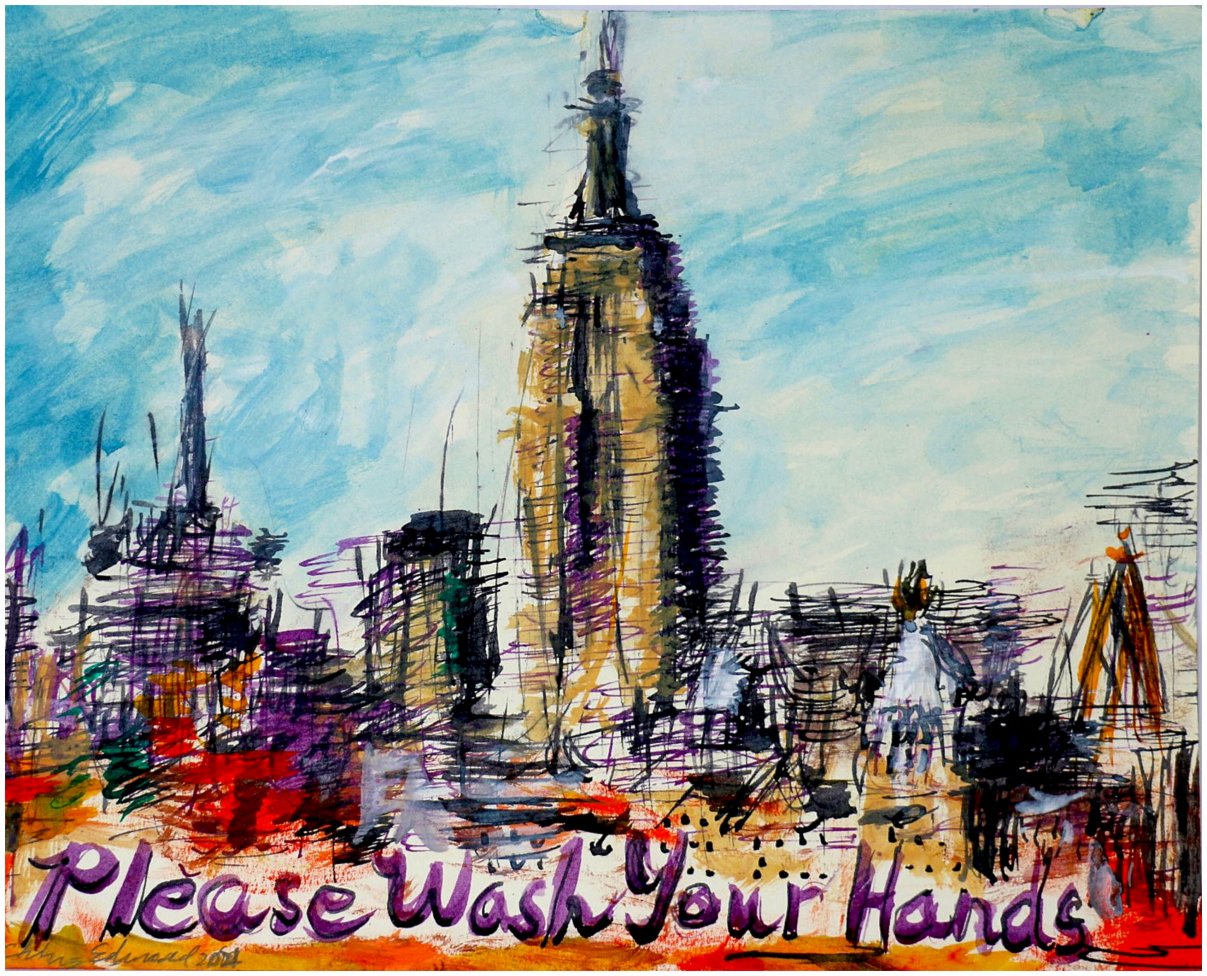
A painting specially created by Edward Ching as an intervention in the Norwegian nursing home ward.

This study is part of a larger study called ‘Hand hygiene, infection prevention and antibiotic use in nursing homes.’ The Regional Committee for Medical and Health Research Ethics (REC), Norway (Ref. 196911 and 226694/REC South-East) and the Norwegian Centre for Research Data (Ref. 118936) reviewed the project. The study took place over 18 months, and during the final 12 months, the nursing ward received three interventions aimed at improving hand hygiene adherence: a UV-light box, multiple posters (Figure 1), and the interviews presented in this article. The ward leader gave us permission to use students to conduct the interviews with the healthcare workers as the third intervention. More information about this study is described in another article (17).

### Design and data collectors

This was an exploratory qualitative study. Both open- and closed-ended questions were used in individual semi-structured interviews with healthcare workers who had direct contact with residents in a nursing home ward. A group of five students in their second year of a bachelor’s degree in nursing volunteered for this study as part of a 3-week course called ‘Research in Nursing.’ The students received 1 week of education provided by two dedicated associate professors. The education included topics on research methods, data collection and analysis, with a critically reflective approach. The students were required to submit a project-based exam to complete the course. The nursing students conducted the individual interviews of the healthcare workers, either in pairs or in groups of three. Most of the students conducted one interview, while one conducted two, and another conducted four.

### Interview guide and conducting the interviews

The students drafted an interview guide, which consisted of 18 questions. It included questions about the healthcare workers’ perceptions of their own and others’ hand hygiene routines, beliefs, knowledge, and thoughts about existing and new interventions to improve hand hygiene adherence. The interviewers practised the interviews with each other before conducting them. The project leader read through the interview guide and wording, and some closed-ended questions were modified. One student was the interview leader and asked most of the questions, while one other student recorded the interviews, made sure that all questions were asked, and sometimes added follow-up questions. The interviews were estimated to take about 20–30 min. The project leader was nearby if they experienced any trouble during the interviews.

### Recruitment and sample

All healthcare workers who worked bedside and wanted to participate were offered the opportunity, with no restrictions regarding occupation. They were recruited on-site, and it was voluntary for all staff members. The sample comprised nine healthcare workers from one nursing home ward. The ward had 20 beds, with 19.5 full-time equivalents and 3.8 equivalent nursing positions. The interviews were conducted over 2 days in March 2022, between 13:00 and 16:00. By choosing this time, healthcare workers from both the day and evening shifts could participate. The staff were encouraged by their leader to participate. After receiving a full explanation of the study and being informed that it would be anonymous, all participants provided signed consent before the interview started. All participants also received chocolate as a thank you for their help.

### Data analysis

One author not participating in the field interviews listened to the audio recordings and transcribed the interviews verbatim. Both authors independently read and analysed the data material. The analytic process was adapted from the systematic text condensation approach described by Malterud (18), which guided the researchers through the process and ensured methodological rigour. Both authors independently identified categories and examples from the transcribed text. Key words and phrases, called ‘meaning units’, were identified, major thematic categories and subcategories were extracted, and then the fit of the categories and meaning units was re-evaluated. The authors compared notes and discussed the findings from their initial analyses to discover agreements and disagreements. This provided a deeper understanding of their interpretation of the empirical data. The authors discussed and summarised the results into four themes. After reaching a consensus on the thematic framework, the analytic researcher reread the data material for a systematic abstraction of meaning units, and based on these units, subheadings emerged. The final thematic categories and example data were discussed (18). We compared our analyses with the students’ analyses from a draft they made before the final exam.

## Results

Nine healthcare workers were interviewed. The interviewees consisted of one nurse, one first-year nursing student, two nursing assistant apprentices, and five nursing assistants. Four major topics—hand hygiene is important, inadequate knowledge, barriers to infection practices, and suggested interventions—and 15 subthemes emerged from the analyses of the transcript data (Table 1).

**Table 1:** The four major topics and subthemes.

Major topics	Subthemes
Hand hygiene is important	Important, stops chain of transmission, not a priority in education
Inadequate knowledge	Guidelines, soap and water or alcohol-based handrub, glove use, protecting themselves
Barriers to infection practices	Shortage of time, stress, tiredness, automatic action
Suggestions for improvement	Reminders, availability, interventions, information

### Hand hygiene is important

All the healthcare workers knew that hand hygiene was important to stop the chain of transmission and agreed that it prevented the spread of infections. They also knew that residents could get seriously ill from infections. One respondent said, ‘I can get an infection from one resident, and just bring it with me to another resident. Then I infect a person who is seriously ill, and then you do not know what happens next’ (i4). Another respondent stated, ‘This ward is well organised to prevent diseases spreading from one patient to another’ (i6). The healthcare workers provided multiple examples in which they needed to conduct hand hygiene, and these situations often revolved around their own or their family’s safety. As one respondent described, ‘I think hand hygiene is important so that you do not take infection with you when you take the bus, and when you go home and are with your family (i3).’

### Inadequate knowledge

Many of the healthcare workers had limited knowledge on when to conduct hand hygiene and did not recall ‘My 5 moments for hand hygiene.’ They also showed limited knowledge of when hand disinfectant was the preferred measure and when handwashing was appropriate. Several of them used handwash and alcohol-based handrub interchangeably and did not differentiate between when each should be used. One respondent said, ‘Usually, I use soap and then alcohol-based handrub; if I’m stressed, I just use alcohol-based handrub’ (i9). Several respondents said that handwash was the best option and their preferred method of hand hygiene: ‘I wash my hands more often than I use alcohol-based handrubs.… I do not usually replace handwashing with alcohol-based handrub. In some cases, I would certainly rather use alcohol-based handrubs’ (i7); and ‘I love washing my hands over alcohol-based handrubs (i3).’

Many said that they often used gloves but were unsure about correct practice, wearing them ‘just in case,’ indicating insufficient knowledge of proper glove use. As one respondent stated, ‘I think it’s easy to use gloves because they are available when you are unsure if you have clean hands or not’ (i3).

Many of the interviewed healthcare workers said that they were good at hand hygiene but ‘the others’ were not as good. This is illustrated in a quote from a respondent: ‘I see that they do not do it [hand hygiene], and I show them. But if they have only been in the room or been with a patient, then it’s something that I do not poke at; I could have done it, but no (i5).’

### Barriers to infection practices

Stress and tiredness were reported as barriers to hand hygiene adherence. The healthcare workers described their work lives as though they had to be in two places at the same time. One respondent said, ‘If you are stressed, it can be a bit like that, I have to be honest, that I can forget completely and that’s it (i9).’ They stated that they had to choose between proper hand hygiene and managing the care of all patients. As one respondent stated, ‘One of them [reason for not performing hand hygiene] is stress… I hear two people getting aggressive in the hallway, and I’m with another patient. I quickly take off my gloves. How am I supposed to manage handwashing and everything when they have already started a fight out there?’ (i4). In the interviews, it emerged that lack of training could serve as a barrier. The training they had received on hand hygiene during their education varied greatly. While most had learned it in their formal training, some said it had been given little importance, while others said it had received a lot of attention. The healthcare workers also stated that they did not receive training in hand hygiene adherence when they started working at the nursing home. Another respondent said, ‘I heard they sent out a video. I have received it but have not seen it’ (i1). Several participants felt that the ward manager should take responsibility. Two also stated that hand hygiene practice was a habit, an automated action on autopilot: ‘If I run around, it goes on autopilot’ (i5), and ‘Hand hygiene is like procedures that you learn, when you study, and so it becomes automatic in a way’ (i2). This highlights how habitual behavior can both support and hinder adherence, depending on whether the habits are aligned with recommended practices.

### Suggestions for improvement

Many said that they needed to be reminded to conduct hand hygiene properly. One respondent suggested ‘reminders, maybe given in the morning and evening report and such’ (i8). Some also mentioned the importance of the availability of hand hygiene dispensers: ‘We have alcohol rub dispensers available inside the room, so once it’s there in the surroundings, it’s just to use them’ (i3).

The healthcare workers had limited suggestions on interventions to improve hand hygiene in nursing homes. Many mentioned the interventions that had already been implemented in the nursing home ward, which they described as ‘fun.’ Many said that the 18 ‘Please wash your hands’ posters from New York City were a good reminder, while one was tired of the posters from the National Institute of Public Health (NIPH). This is illustrated in their quote: ‘It’s [the New York City posters] a very good reminder, because you often see it as soon as you come out of the patient’s room. … I’m tired of those posters from NIPH on the walls all the time (i4).’ One respondent said that she got scared before trying the UV light box for the first time because she was afraid of discovering that she did not conduct hand hygiene as well as she should: ‘Therefore, I believe in the machine [UV-light box]. Maybe we will improve, and hand hygiene will improve, because everyone sees and is scared’ (i1).

Another respondent suggested that they could attend a course in hand hygiene, stating that ‘from time to time, we are offered a course, but not in hand hygiene’ (i6).

## Discussion

To the best of our knowledge, this is the first study allowing bachelor students to interview healthcare workers about hand hygiene in nursing homes as an intervention to improve hand hygiene adherence. An interesting finding in this study is that several of the respondents said that other healthcare workers did not perform hand hygiene when it was required. Even so, few of them wanted to remind ‘the others’ about how hand hygiene should be performed correctly. The findings show a line between ‘them’ and ‘me’—‘They do not do it properly,’ and ‘I do it pretty well’—which was also found by Løyland, Wilmont, Hessels, and Larson (16). This may be because most people desire to be the ‘best in class.’ We can also speculate about whether it is their own inadequate knowledge that prevents them from speaking up, or whether there is a culture in the department that this is not the individual’s responsibility. One possible explanation for why healthcare workers dis not comment on others’ lack of hand hygiene adherence may be rooted in the power dynamics within healthcare. Individuals with higher education and status often hold more influence, making it challenging for those with lower status to speak up. Effective collaboration within healthcare teams has been identified as a critical factor in reducing adverse events and enhancing patient safety, highlighting the importance of addressing hierarchical barriers to open communication (19).

The healthcare workers in this study indicated that they used gloves in certain situations as a precautionary measure. This suggests a lack of understanding regarding the correct usage of gloves, as well as the specific situations in which they should be used. Other studies have also shown that the use of gloves decrease hand hygiene adherence (3, 20). Most of the healthcare workers explained that proper hand hygiene was important for preventing the spread of infections. At the same time, they lacked knowledge on when and how they should conduct it. Other studies have shown that some healthcare workers have good hand hygiene adherence, and that this is highly dependent on profession (3, 5). Nurses in long-term care facilities for older adults continuously strive to balance working hygienically with creating a homelike environment (12, 15).

The healthcare workers had some suggestions and remarks about what they thought affected hand hygiene in the facility. Availability was said to be important, and this was also found to be the only intervention with a moderate effect in a Cochrane review (7). Since this ward was implementing interventions for improving hand hygiene, many healthcare workers mentioned the interventions they had experienced. One such intervention was conducting hand hygiene under UV light, which made some healthcare workers feel that their hand hygiene was not good enough. Most said that they wanted reminders to conduct hand hygiene. Several noted that the posters hung as an intervention were a good reminder. One stated that they were tired of the posters made by NIPH. This may indicate that posters will not have a long-term effect if they are not changed regularly. This finding is supported by other studies that have found a short-term effect of interventions for improving hand hygiene (6, 7, 21). Several stated that hand hygiene action was performed automatically, and this statement illustrates the difficulty in improving an action that is carried out on autopilot. Nursing home leaders should take responsibility for implementing targeted training programs for healthcare workers, introducing regularly updated reminders throughout the year, and ensuring that hand hygiene disinfectants are available at points of care.

Having nursing students conduct the interviews may have had both advantages and challenges. The students themselves were enthusiastic about conducting the interviews. They told the project leader that it was exciting to participate in “real” research and that they learned a lot about how to conduct interviews. This aligns with findings from another study where nursing students participated in research by observing hand hygiene adherence in nursing homes. The students wrote in their reflection notes that participating in research increased their essential knowledge (5). In this study, there were some differences in how the students conducted the interviews. Some students strictly followed the interview guide, while others posed several additional relevant questions. Some were leading questions, which should be avoided in an interview setting (22), although they are hard to avoid completely. Having inexperienced nursing students conducting the interviews may convey less authority, potentially leading interviewees to respond with greater confidence and provide less conforming answers (22). This combination may have influenced the answers from the healthcare workers to varying degrees.

### Strengths and limitations

This study has both strengths and limitations. It is a strength that this is the first study to interview healthcare workers in nursing homes in Norway about their hand hygiene practices and their thoughts on improving hand hygiene. Additionally, the use of bachelor students to conduct the interviews has, to our knowledge, never been done before in nursing homes in Norway, and the students benefited from increased knowledge by participating. There are also some limitations in the study. First, those who were interviewed volunteered to participate, and as in other studies, it is possible that those volunteering differed from those who did not volunteer or have time to participate. Second, it is possible that the answers from the healthcare workers were influenced in unknown ways. Some responders were nervous and felt that the interviews were a type of control. Interviewees often want to provide the ‘right’ or socially desirable response, and some of the healthcare workers said that they wanted to answer the questions correctly. Third, the interviewers were nursing students with limited experience conducting interviews.

## Conclusion

Five students in their second year of a bachelor’s degree in nursing conducted the interviews. Students can learn a lot from being involved in research, but it is important to ensure that they have sufficient knowledge of the principles of research ethics to protect the integrity of the respondents. These interviews were used as an intervention to increase hand hygiene adherence in a nursing home ward. The healthcare workers said that it was challenging to balance good care for residents with proper hand hygiene, even though they knew the importance of hand hygiene for preventing the spread of infections. The healthcare workers reported barriers to proper hand hygiene as being stress and tiredness. They also had inadequate knowledge for when to conduct hand hygiene and did not receive training. They had limited suggestions for interventions to improve hand hygiene in nursing homes, but they emphasized the importance of availability. Nursing home leaders should ensure healthcare workers receive proper training, regular reminders and have easy access to hand hygiene resources.

## Data Availability

The raw data supporting the conclusions of this article will be made available by the authors, without undue reservation.
